# Re-Processing of Multilayer Plastic Materials as a Part of the Recycling Process: The Features of Processed Multilayer Materials

**DOI:** 10.3390/polym12112517

**Published:** 2020-10-28

**Authors:** Ville Lahtela, Shekhar Silwal, Timo Kärki

**Affiliations:** 1 LUT Re-Source Research Platform, LUT University, P.O. Box 20, FI-53851 Lappeenranta, Finland; 2 Fiber Composite Laboratory, LUT University, P.O. Box 20, FI-53851 Lappeenranta, Finland; shekhar.silwal@student.lut.fi (S.S.); timo.karki@lut.fi (T.K.)

**Keywords:** packaging plastic, multilayer plastic, re-processing, recycling, properties, sorting

## Abstract

The weight of packaging materials will be increased with advanced innovations, such as multilayer plastic. The consequence of the advanced innovations is challenges in the following reuse activities. This study aimed to investigate the properties of multilayer plastic materials after recycling processes and will increase the awareness of plastic packaging material for reuse options. In this research, the materials were produced from food packages by crushing them and treating them with injection molding equipment. The implementation of materials in the processing was tested, and the structural and mechanical characteristics of the produced plastic materials was evaluated and discussed. Based on the completed tests, plastic materials used in food packages have the clearest differences in the material features, for instance, the melt flow rate and elongation rate in the tensile test that varied between 2.96–48.4 g/10min and 2–289%, respectively. The variation in the characterizations ranged widely between the material structures. The results indicate that solid plastic packaging materials have better mechanical features compared to foil materials. The structural analysis of materials showed that multilayer plastic includes a wide spectrum of different elements within materials, creating a challenge for future recycling.

## 1. Introduction

Plastic is an excellent material with outstanding properties, but if it is carelessly processed it can cause remarkable global concerns for humans and the environment. For example, under exposure to solar radiation, plastic material releases a variety of chemicals during degradation, such as greenhouse gases, methane ($CH_{4}$) and ethylene ($C_{2}H_{4}$) [1]. Additionally, plastic from waste electrical and electronic equipment (WEEE) might include some flame retardants [2] and municipal solid waste (MSW) might include waste plastic with a high heavy metal content [3], which also raises concerns for the environment. Due to the forecast increase in the population [4], the use of plastic is expected to grow, as is the amount of plastic debris [5]. The increasing amount of plastic waste causes concerns for global non-sustainable development, for example in coastal areas, where in the worst-case scenario, waste plastic might be able to enter the ocean [6]. It is predicted that plastic marine debris will increase if waste management infrastructure improvements are not made [7]. Therefore, plastic waste should be recycled and re-utilized in a controlled manner and it is the responsibility of all of us to promote this topic.

The influence of the plastic industry on society is remarkable. For example, in Europe, the distribution of global plastics production is just 17%. Nonetheless, the turnover of the European plastic industry has been reported to be more than EUR 360 billion, and it employs more than 1.6 million people [8]. Due to plastic industry’s significant waste issues, the principles of circular economy must be taken into account in their actions. The European Commission (EC) launched a plastic strategy that boosted demand for recycled plastics and will enable us to make the plastic sector more circular and resource-efficient [9], which was supported by industry [8]. Regarding recycling, only a minor percentage (14–18%) of all plastic materials was recycled at a global level in 2018 [10]. According to estimates, 17% of all packaging films are made of several components, creating what is known as a multilayer material [11,12] which is marked with the symbol “7” and “other” resin identification code (RIC). The previously mentioned estimation is fairly congruent with the study by Dahlbo et al. [13], where the share of the monotype plastics in MSW plastic fractions was estimated to be 80%. The growth of multilayered plastics is expected to be about 7% [14] and during the years 2018–2026, the global market value is forecasted to expand by 4.4% with CAGR (compound annual growth rate) [15]. Multilayer materials improve the shelf life and stability of the product during storage due to, for example, better barrier properties with a low thickness (200 µm) [16]. The previously mentioned information demonstrated that multilayer plastics have a significant role in the future. Multilayer plastics typically consist of six material layers on average, including several polymers. The minor amounts of ethylene vinyl acetate (EVA), ethylene vinyl alcohol (EVOH), and prints are also included in the structure of multilayer plastic [13]. Typical manufacturing technologies for multilayer materials are lamination and co-extrusion [17]. In the case of co-extrusion, the different plastic materials are melted through an extruder, and then co-extruded layers bond directly to each other. In some cases, a so-called extrudable adhesive might be applied between materials as a third polymer, such as anhydride-modified polyethylene (PE) or anhydride-modified EVA. In the case of non-plastic materials, the lamination process might be the most general manufacturing method [18].

In total, 32.5% of European post-consumer plastic was recycled in 2018, and over 80% of this was recycled inside the European Union (EU). However, too large a share of European waste plastic will end up in energy recovery and landfill; 42.6% and 24.9%, respectively. The recycling option is better known in the case of plastic packaging waste, where the recycling rate increased from 42% to 92% over the time period from 2006 to 2018 [8]. Plastic materials have a high recyclability potential, so the recyclability of multilayer material, for which the current share is about a fifth of all plastic fractions, should be reviewed with care, especially from the viewpoint of upstream lifecycles [11,12,13,19]. However, the global recycling rate is remarkably low compared to this European value; only about 14% of plastic packaging production is recycled worldwide [20]. The potential to increase recycling is still huge, and according to the estimation, inter alia, food packaging plastic applications have the highest recycling potential in existing facilities [21]. Even though plastic recycling requires action, the polymers under the category ‘others’ (RIC 7) are undesirable plastics for recovery centers due to the difficulties in recycling [22]. Monotype plastics are more suitable for mechanical recycling [13]. Plastic features and quality will greatly affect recyclability. A small amount of unfavorable material can decrease the value of the end-product, which might manifest as poorer adhesion or changed color [19]. Plastic polymers should be separated and sorted in order for recycling to be more effective and useful. Therefore, in finding solutions for novel recycling routes, it is necessary to ask which will contribute to the recycling option instead of energy recovery or landfill, also in the case of multilayer materials that have a major contribution in the future.

The recycling of multilayer materials could be a multi-step process, starting with a collection and sorting of materials. It is essential to identify the composition of consumer plastic materials in order for effective recyclability to be achieved. After identification, multilayer materials can be processed by various technologies, such as chemical or mechanical recycling processes. Polymer material was degraded to smaller molecules in the chemical recycling processes, which might include various technologies, inter alia, chemolysis, pyrolysis, fluid catalytic cracking, hydrogen technology, or gasification. Correspondingly, mechanical recycling processes might also include several process phases, such as shredding, sorting, cleaning, extrusion, filtering, or pelletizing. Plastic waste can be separated by utilizing optical sorting, such as spectroscopic identification and high frequency cameras [23].

As mentioned, the amounts of plastics will be increased in the future, due to the forecasted increase in the population and trending e-shopping culture. Ergo, the multilayer material should also be recycled in future. The aim of this study was to assess the feature of multilayer plastic materials after recycling processes and evaluate its reusability as a material in novel applications, together with other plastic materials. A minor amount of publication from the context of multilayer plastic material was published, addressing a need for this kind of novel study. Research into multilayer recycling is essential because it creates new openings in the research the research theme and the option to develop novel innovations into markets. The individual materials are identified and separated, and the functionality of material is discussed. The key research question is: how can single-use multilayer materials be processed and reused as a raw material? Extensive studies of the processing of multilayer materials have not been made, but we addressed a general need for the discussion of this topic.

## 2. Materials and Methods 

**Figure 1:**
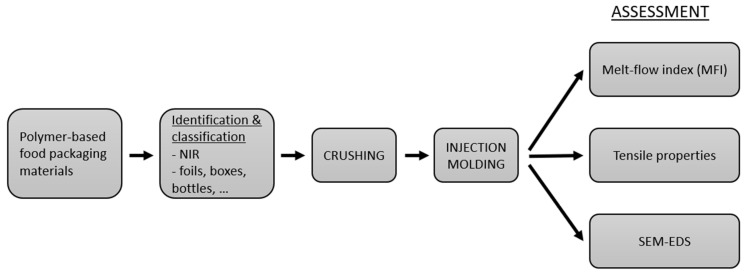
A process diagram of the study.

The processing of multilayer materials started with collection and sorting steps, where the material was identified properly. After thorough classification, the material was processed by crushing and injection molding technologies. The material features were analyzed from the processed materials by three main tests, namely, melt flow index (MFI) test, tensile properties test and, to study the material structure, scanning electron microscope with energy dispersive spectroscopy (SEM–EDS). The description of the studied process is illustrated in the following Figure 1.

### 2.1. Collection and Identification

**Figure 2:**
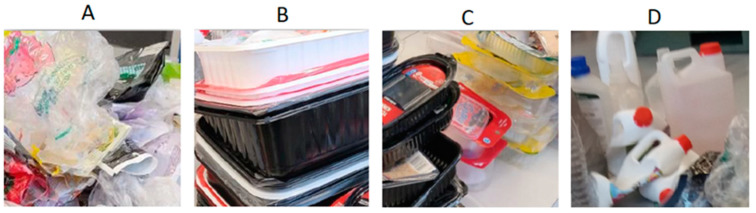
Illustrative examples of foils (**A**), boxes (**B**), slide boxes (**C**), and bottles (**D**) which were used in this study.

In this study, the multilayer materials consisted mostly of the packages used for food packaging, which were collected from a student restaurant and from households. In addition to the multilayer material, the study also includes other identified polymer-based materials, such as boxes and bottles. According to the intended use, the materials were initially classified into foils, boxes, slide boxes, and bottles. The examples of materials are presented in Figure 2.

In addition to the intended use, the initially classified materials were identified based on the material polymers. The identification of material is an important task which helps to avoid the contamination of different materials and improve the quality of material in further stages. Identification can be done by various technologies, such as with a spectroscopic instrument. In this study, plastic materials were classified according to the RIC symbol of the polymers. For plastic materials without a code symbol, polymer composition was analyzed by portable near infrared (NIR) spectroscopy equipment (Thermo Scientific microPHAZIR PC, Thermo Fisher Scientific, Waltham, MA, USA), which identifies material in the spectral range of 1600–2400 nm. After the identification, material amounts were measured by weight. 

### 2.2. Crushing

The identified polymer materials must be granulated to conduct further processing. The materials were crushed into small pieces with a Shini SG-1635N low-speed granulator (Shini Plastic Technologies, Inc., New Taipei, Taiwan), equipped with a 5.00 mm sieve.

### 2.3. Injection Molding

The crushed granulate materials were further processed by injection molding process, in which the materials were heated, melted and mixed thoroughly with a rotating screw. The process was performed with an injection molding machine BOY 30 (Boy), with the following parameters: melt temperature 170 °C, injection pressure 6.9 MPa, and injection time 3 s. The 15 samples of each material were manufactured for further analysis.

### 2.4. Analysis of the Properties of the Studied Multilayer Materials

#### 2.4.1. Melt Flow Index (MFI)

The viscosity and melt flow rates of the studied material in a molten state were identified. The melt index of the studied materials was measured with the laboratory melt indexer (Dynisco LMFI-2NENNNN, Franklin, MA, USA) based on the standard ISO 1133-1 method A/B. The MFI test gathered information about the flowrate of the studied materials. The melt flow rate (MFR) and the melt volume flow rate (MVR) were recorded as an average of three tests.

#### 2.4.2. Tensile Properties

The reaction of materials to resist force under tension was analyzed by a mechanical property test. The tensile properties (strength and modulus) of the injection molded materials were analyzed with a testing apparatus Zwick Z020 (Zwick Roell group), according to the ISO 527-2 standard. Testing conditions were congruent for all materials, without any differences in material composition. The information about the ultimate tensile strength, tensile elongation, elastic limit and tensile modulus of elasticity were recorded. The test samples were conditioned in a chamber where the temperature and humidity were set to 23 °C and 50%, respectively.

#### 2.4.3. SEM–EDS Analysis

The surface morphology of the materials was studied by SEM–EDS analysis. A scanning electron microscope with energy dispersive spectroscopy (SEM–EDS) apparatus (Hitachi SU3500, Tokyo, Japan) was used to examine the materials at an accurate level. The apparatus produced detailed high-resolution images from the sample and analyzed its elemental distribution with the following operation conditions: voltage 15.0 kV, vacuum 90 Pa, and magnification varying between 50 and 500.

## 3. Results

The materials were classified and identified based on the use and polymers identification group, and the results are presented in Table 1. The most general plastics were PE, polypropylene (PP), and polyethylene terephthalate (PET), identified with RIC or NIR spectroscopy. Unidentified plastics, such as dark materials, were sorted into a group labeled ‘other’ (RIC 7).

The identified materials were processed separately. They were divided into seven categories; PP-box, PP-bottle, PE-bottle, PET-bottle, PP+PE-foil, 7-foil, and 7-box, as mentioned in Table 2. The division was made based on the combination of material application and the identified polymer, such as PET, PE, PP, and other (7).

### 3.1. MFI Analysis

The MFI test was performed at a temperature of 230–275 °C with a weight of 2.16 kg, except for the material group of the 7-box, where a higher weight (5.00 kg) was needed. The more detailed conditions for materials are shown in Table 2, with the test results of the MFI tests.

The results demonstrated that the groups of PET-bottles and 7-box have relatively high MVR and MFR values, especially when the higher treatment temperature (275 °C) is taken into account in the properties test. In addition, the PP-bottle group has a high value while that of the PP-box group has a low value, indicating the remarkable influence of additives within polymers. The lowest value of the PP-box indicates that this material has a less viscous nature than other materials. Overall, foil materials were more viscous compared to similar solid material, indicating easier processing in the challenging productions.

After MFI analysis, the material was molded with an injection molding process to create material specimens for further analysis. Materials were processed successfully with the standard parameters (see Section 2.3 Injection Molding), as expected for the polymer of PET. Due to the different polymer nature, the outcome of the PET polymer was not as expected, and its molding was a failure, also with varied parameters. Therefore, the results from the PET-bottle group are not available in further tests.

### 3.2. Tensile Properties

**Figure 3:**
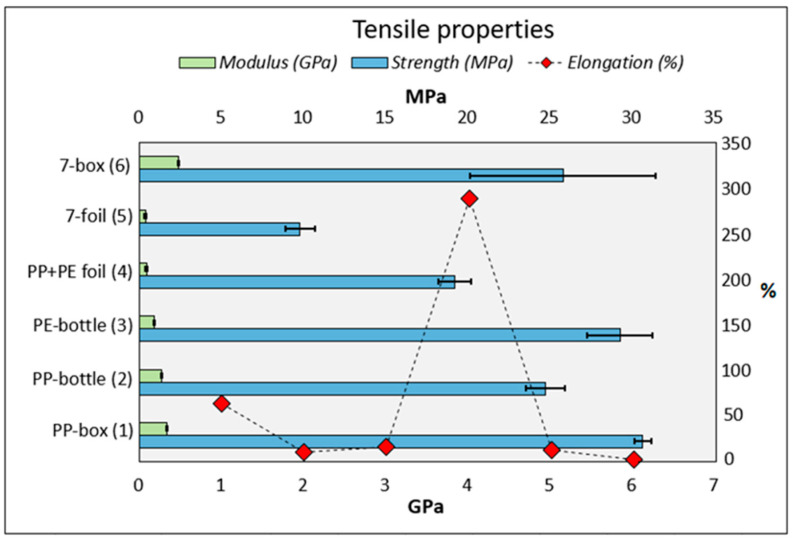
The results of tensile strength and modulus as clustered bar charts with the standard deviations as error bars, presented in the x axis. Light-blue clustered bar charts represent the tensile strength and light-green clustered bar charts represent tensile modulus results. The elongation at break values are shown with a red square marker with straight lines in the right-hand *y* axis.

**Figure 4:**
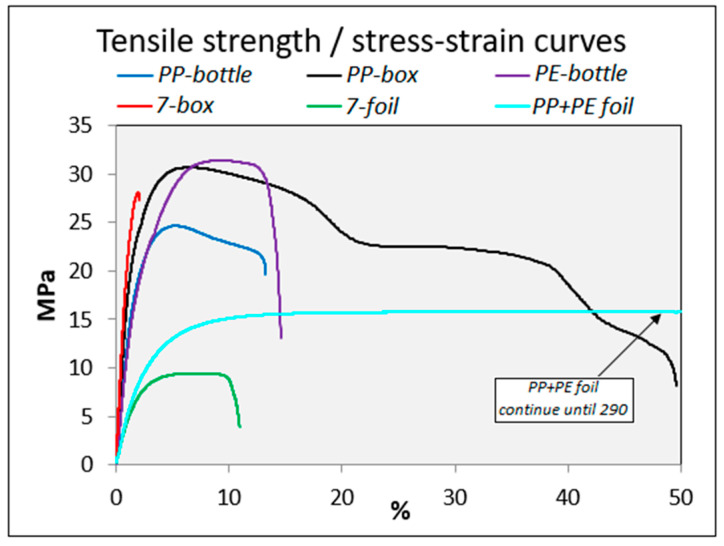
Stress–strain curves of the studied material, as an example, under the tensile properties test. Stress–strain curve of the PP+PE foil material continuing until an elongation of ~290%.

Tensile properties were analyzed according to the standardized test. The test sample is clamped between the cross head and the movable head jaws. It is then stretched along the major longitudinal axis with constant elongation rate until the test sample breaks. The test primarily measures the amount of force applied and the elongation of the sample. The values obtained from the test are presented in the form of clustered bar and stress–strain curve charts in Figure 3 and Figure 4, respectively.

The more solid materials (boxes and bottles) have better tensile properties compared to the thinner and flexible materials (foils). For example, the strength of solid multilayer material (7-box) was over twice as high (2.63 MPa) compared to that of multilayer foil (7-foil). The results show that the material of PP+PE foils has a very high elongation at break value. The elongation at break value of the 7-box was only 2.35%, which is very low compared to the other ones. For example, the elongation at break values of the PP-box and PP+PE foil groups were 64.24% and 289.02%, respectively. Therefore, final break failure with foil material takes a long time.

### 3.3. SEM–EDS Analysis

Two specimens were studied from each of the six groups of materials (Table 3). A closer view of the surface with an SEM device helped to find cracks and fractures from the samples and the presence of some impurities on the surfaces, but clear differences between the materials were not found. However, energy dispersive spectroscopy analyzes the chemical constituent of materials. Overall, carbon is the clearest biggest element in the studied material but in the other material groups (RIC 7), nitrogen was also included in a high concentration. The other elements present in all the groups are due to the additives or other foreign impurities present in the material. The 7-foil and 7-box groups included a wide spectrum of different elements compared to the other materials, such as the presence of chlorine, which seems to be quite an unusual material in plastic packaging. The volume of chlorine is higher for 7-foil material compared to 7-box material, which might be due to the bonding substance. The multilayer material layers were bonded by using different additive layers and the presence of numerous elements, which might also be a reason for the presence of chlorine.

## 4. Discussion

This study showed that the plastic waste stream includes various polymer groups, which were used for various purposes. A large volume of multilayer materials with unknown polymer groups were also included in the waste stream, consisting mostly of packaging materials, the recycling of which might cause some challenges. The possible recycling methods for polymer-based multilayer packaging has been previously studied, and two possible options, delamination or compatibilization, were proposed for the recycling of multilayer materials. In the delamination process, all layers were delaminated and separated but the process is suitable only for limited amounts of multilayer material, without economic considerations. The compatibilization step was also not an effective method due to the fluctuations of material composition [24].

The PET polymer causes some challenges in the material test which were not solved with a higher processing temperature (275 °C) than the universal melting point of polymer (260 °C). The process might cause a failure for polymers if the barrel temperature of the injection molding machine is not uniformly heated. In this case, the polymer might cool quickly after melting, which can block the nozzle. The material might also cause some challenges because of the nature of PET polymer, which is hygroscopic, so it is easily available for water molecules at melt processing temperature. According to previous literature, successful results with PET cannot be expected because the limited crystallization without nucleating agents caused poor mechanical properties [25]. In addition to the presence of nucleating agents, the problem might be avoided by drying the polymer before the injection molding process. The PET polymer is slow to crystallize and tends to become brittle during crystallization, and it also has a low glass transition temperature, which needs adjuvants for enhancements. PET within blends have weaker properties compared to the individual PET [26], so none of the excellent properties will disappear without the presence of PET. Additionally, 80% of the recycled PET bottles are turned into polyester fibers for lower-grade products [19]. Some other technologies might be more useful for more challenging polymers, such as PET, when the mixing of materials could also be verified more exactly.

The strength properties of the tested materials were found to be higher for solid materials than foil materials. The solid other materials (7-box) also have features that are comparable to other solid materials. However, in the case of foils, the strength of the material group of foil (7-foil) was reduced compared to the other ones (PP+PE foil). Despite the good strength of the 7-box, it has the lowest elongation at break value, which indicates that the deformation feature is weak, and the material is ductile and brittle. Overall, the strength features of recycled materials were impaired, but this could be improved by the mixing of virgin materials. For example, the increasing content of PP in PE improved the tensile strength of the composite [27]. Testing parameters were identical for each material in the strength test in order for the comparability between materials to include none of the variables. The results from the MFI tests did not correlate strongly with the mechanical strength because the highest and lowest MFI results caused a certain best strength result. The mechanical properties of the multilayer material might be directly dependent on the dominant polymer in the blend.

The presence of chlorine in the materials of the 7-foil and 7-box might be explained by the polyvinyl chloride (PVC), if it is used in the manufacturing. Some other elements can be from the additives and stickers that were used in the packages of multilayer materials. For example, sodium chloride (NaCl), calcium carbonate ($CaCO_{3}$) silicon oxide ($SiO_{2}$) and wollastonite minerals ($CaSiO_{3}$) are major additives used in packages, which explains their presence [18]. The SEM examination has shown that the obtained impurities on the samples´ surface addressed the need to clean materials before processing, especially in the case of multilayer materials. A solution for cleaning could be a hot caustic pre-wash that removes labels and contaminations [28].

Despite the accepted properties of multilayer materials, the separation of material layers must be studied in more detail in the future, and results might be found in the field of chemical recycling applications. A certain solution for the separation of layers could be the utilization of different solubility properties which could split laminated layers. For example, polyurethane adhesives can be dissolved in formic acid [18]. In addition, multilayer materials should also be analyzed under various applications because limited studies have been performed under different environmental conditions.

## 5. Conclusions

Various polymer-based materials with rapidly expanding applications in the field of packaging materials have a huge economic influence and high recycling potential. Certain properties of multilayer plastic might cause challenges during the secondary processing steps. This work studied the reusability options of multilayer plastic material as part of the recycling process, performing the examination of material properties with the test procedures. The experiments confirmed that materials have specific properties, depending on the use and applications. The melt flow properties varied between the same polymer, depending on the applications (box vs. bottle). The strength property test indicated a significant diversity between solid and foil materials. The dominant polymer will form a matrix, so the miscibility of the final blend will influence the mechanical properties. The unique nature of multilayer plastics compared to other plastics was revealed in the SEM–EDS test, in which the composition of multilayer plastic proved to be wide, including 10 and 13 identified elements while other ones include six or less identified elements. Previously, it has generally been known that polymer sorting and separation is useful from the viewpoint of material properties, and this study proved the same with multilayers. The processing method of multilayer materials is to date generally unavailable, creating a need to continue this kind of research in the future. Considerably more work will need to be done to determine how multilayer material could be sorted from other plastic and how its material composition could be separated into polymer levels. The specific collection of multilayer material might be a solution for the quality re-processing but probably, the development of sorting technologies could be the more cost-effective way to re-utilize multilayer plastic as a part of recycling process. 

## Figures and Tables

**Table 1:** The recognized multilayer materials with measured weights in grams (g).

Polymer	Foil	Box	Slide box	Bottle
PET (1)	-	-	-	486.6
PE (2 & 4)	105.3	76.70	-	696.6
PP (5)	37.90	1043	44.40	353.4
OTHER (7)	200.8	214.5	527.0	-

**Table 2:** The melt flow index test conditions and results.

Material	Conditions		Results	
	Weight (kg)	Temperature (°C)	MVR (cc/10 min)	MFR (g/10 min)
PP-Box	2.16	230	4.42	2.96
PP-Bottle	2.16	230	32.5	19.7
PE-Bottle	2.16	230	8.18	3.27
PET-Bottle	2.16	275	36.7	48.4
PP+PE Foil	2.16	230	7.39	5.34
7-foil	2.16	275	18.1	12.9
7-box	5.00	275	46.4	22.3

**Table 3:** The elemental composition of the multilayer plastic materials. Amount shares are given as an average of the weight percentage (wt %) from the whole mass of the studied sample.

Polymer	PP-Box	PP-Bottle	PE-Bottle	PP+PE Foil	7-Foil	7-Box
Carbon	99.1	99.1	99.1	99.4	77.8	77.7
Nitrogen	-	-	-	-	10.2	21.0
Oxygen	-	-	-	-	8.90	-
Sodium	-	-	-	-	0.13	0.08
Magnesium	0.03	0.05	-	-	0.02	0.04
Aluminum	0.30	0.62	0.28	0.21	0.37	0.62
Silicon	-	0.11	0.06	0.05	0.10	0.10
Sulphur	-	-	-	-	0.04	-
Chlorine	-	-	-	-	1.26	0.07
Potassium	-	-	0.04	0.06	0.17	0.09
Calcium	0.22	0.11	0.12	0.05	0.09	0.14
Titanium	0.13	-	0.39	0.28	0.84	0.16
Palladium	-	-	-	-	0.00	-
Barium	0.26	-	-	-	-	-
